# Psychiatric Adverse Drug Reactions and Potential Anti-COVID-19 Drug Interactions with Psychotropic Medications

**DOI:** 10.22037/ijpr.2021.114717.15007

**Published:** 2021

**Authors:** Parisa Ghasemiyeh, Negar Mortazavi, Iman Karimzadeh, Afsaneh Vazin, Laleh Mahmoudi, Ebrahim Moghimi-Sarani, Ashkan MohammadSadeghi, Mina Shahisavandi, Ali Kheradmand, Soliman Mohammadi-Samani

**Affiliations:** a *Department of Clinical Pharmacy, School of Pharmacy, Shiraz University of Medical Sciences, Shiraz, Iran. *; b *Pharmaceutical Sciences Research Center, School of Pharmacy, Shiraz University of Medical Sciences, Shiraz, Iran. *; c *Department of Psychiatry, Research Center for Psychiatry and Behavioral Sciences, Shiraz University of Medical Sciences, Shiraz, Iran. *; d *Department of Psychiatry, Taleghani Hospital Research Development Committee, Medical School, Shahid Beheshti Medical University, Tehran, Iran. *; e *Department of Pharmaceutics, School of Pharmacy Shiraz University of Medical Sciences, Shiraz, Iran.*

**Keywords:** COVID-19, Psychotropic medication, Adverse drug reactions, Drug-drug interactions, Pharmacokinetics

## Abstract

*Coronavirus* disease 2019 (COVID-19) management in patients with predisposing psychiatric disorders would be challenging due to potential drug-drug interactions (PDDIs) and precipitation of their disease severity. Furthermore, COVID-19 itself might precipitate or induce unpredicted psychiatry and neuropsychiatry complications in these patients. In this literature review study, the psychological impacts of COVID-19 and major psychiatric adverse drug reactions (ADRs) of COVID-19 treatment options have been discussed. A detailed Table has been provided to assess potential drug-drug interactions of COVID-19 treatment options with psychotropic medications to avoid unwanted major drug-drug interactions. Finally, potential mechanisms of these major drug-drug interactions and possible management of them have been summarized. The most common type of major PDDIs is pharmacokinetics. Hydroxychloroquine/chloroquine and lopinavir/ritonavir were the most involved anti-COVID-19 agents in these major PDDIs.

## Introduction

In late December 2019 new coronavirus was first detected in Wuhan city of China (1). Now, this novel coronavirus (2019-nCoV), also called severe acute respiratory syndrome *coronavirus* 2 (SARS-CoV-2), has become a pandemic, and many people around the world are currently suffering from this disease. *Coronavirus* disease 2019 (COVID-19) can be presented with respiratory signs and symptoms including cough, chills, fever, rhinorrhea, dyspnea, sneezing, hypoxia, chest pain, and gastrointestinal adverse reactions including nausea, vomiting, diarrhea, and abdominal pain. Other common signs and symptoms of this disease are myalgias, fatigue, ageusia, and anosmia (2, 3). Other important extra-pulmonary presentations of COVID-19 are thrombotic events, acute kidney injury (AKI), cardiac dysfunction as well as an acute coronary syndrome (ACS), dermatologic disorders, and hepatic injury (4). Also, COVID-19 could be presented with many neurologic (5, 6) and adverse psychiatric reactions (7, 8) that should be considered by clinicians and health care providers. Also, COVID-19 pharmacotherapy would be challenging in patients with predisposing neurologic and psychiatric disorders regarding potential drug-drug interactions (PDDIs) and precipitation of adverse drug reactions (ADRs) (6, 9-12). Till now, many drugs have been considered in COVID-19 management. In this study, different COVID-19 treatment options, based on the latest version of the Infectious Diseases Society of America (IDSA) guideline, have been classified to 1) therapeutic options that recommendations are against their use, 2) therapeutic options that recommendations are in favor of their use, 3) therapeutic options that their uses are allowed only in the context of clinical trials, and 4) therapeutic options that are under evaluation (13). 

## Experimental

Literature was searched on Scopus, PubMed, Web of Science, Google Scholar, Up-to-date^®^, and Liverpool^®^ databases by using the key search terms including “COVID-19”, “SARS-CoV-2”, “psychotropic medication”, “psychiatric ADRs”, and “PDDIs” from 1950 until December 2020. In this regard, the first titles and abstracts of the published papers were reviewed for initial screening, and then relevant articles were selected and reviewed. Firstly, in this review, the psychological impacts of COVID-19 and major psychiatric ADRs of COVID-19 treatment options have been discussed. Also, potential pharmacokinetic DDIs have been considered. A detailed table has been provided for PDDIs of each COVID-19 treatment options and psychotropic agents. Finally, potential mechanisms of the major DDIs and possible managements of them have been discussed.

*Psychological impacts of COVID-19*

The catastrophic COVID-19 pandemic has appeared since late 2019 (14). This virus has a high potential for human-to-human transmission. It may cause a severe and fatal illness characterized by acute respiratory distress syndrome, multi-organ failure, and death (14). Besides, this virus has different psychiatric and neuropsychiatric manifestations. According to the systematic review of two previous outbreaks, including severe acute respiratory syndrome (SARS) and the Middle East respiratory syndrome (MERS), among the participants, the most common psychiatric symptoms at the acute phase of illness were confusion (27.9%), depressed mood (32.6%), anxiety (35.7%), impaired memory (34.1%), and insomnia (41.9%) (15). In the post-illness stage of these outbreaks as mentioned earlier, the most commonly reported psychiatric reactions were depressed mood (10.5%), insomnia (12.1%), anxiety (12.3%), irritability (12.8%), memory impairment (18.9%), fatigue (19.3%), traumatic memories (30.4%), and sleep disorder (100%) (15). A recent study on 153 cases of COVID-19 revealed that 62% of the recruited patients had at least one cerebrovascular event. Among these reported cerebrovascular events, ischemic stroke (74%), intracerebral hemorrhage (12%), CNS vasculitis (1%), altered mental status (31%), unspecified encephalopathy (23%), and encephalitis (18%) were the most common neurologic features (16). The role of pathological inflammation in CNS associate with new coronavirus infection can promote this hypothesis that in addition to pandemic-associated psychological distress, the direct effects of the virus itself, severe acute respiratory syndrome, and the subsequent host immunologic response can lead to neuropsychiatric demonstrations (17). On the other hand, there are psychosocial risk factors for incidence of psychiatric disorders during the COVID-19 pandemic that include fear of family members getting infected, frequent exposure to infected persons, limited access to testing and medical care for COVID-19, work overload for health care workers and job losses and economic insecurity (18, 19). COVID-19 patients indicate high levels of Interleukin (IL)-1β, IL-6, Interferon (IFN)-γ, CXCL10, and CCL2 suggesting activation of T-helper-1 cell function. In addition, in COVID-19 elevated levels of T-helper-2 cell-secreted cytokines (such as IL-4 and IL-10) were appeared (20). The high concentration of some cytokines such as IL-1β, IL-6, IL-10, IFN-γ, TNF-α, and transforming growth factor-β (TGF-β) are known to be involved with the incidence of psychiatric disorders (21-24). Besides, hypercoagulation and cerebrovascular events in patients with severe infection can cause both increased blood clots and bleeding, potentially causing ischemic and hemorrhagic stroke (17). These factors, including elevated cytokine concentration and coagulation disorder, can lead to potential direct and indirect causes of long-term cognitive impairment. 

On the other hand, possible medication therapies for the treatment of COVID-19 also result in neuropsychiatric side effects. Hydroxychloroquine and chloroquine have drawn considerable attention for their potential to treat *coronavirus* disease; however, new evidence is against the use of these medications because of their safety concerns (25). Hydroxychloroquine and chloroquine can cross the blood-brain barrier and achieving concentrations 10–20 times greater than plasma concentrations. Neuropsychiatric clinical manifestations of hydroxychloroquine and chloroquine are reported in many studies include agitation, bradyphrenia, delirium, disorientation, irritability, nervousness, adjustment disorder, confusion, and suicide attempt (26). Besides hydroxychloroquine, IFN-beta as other recommended treatment for COVID-19 (27) indicates psychiatric disorder, especially depression as an ADR and patients with a history of depression or suicide attempts should be under meticulous observation during the time of therapy (28-30).

In hospitalized patients, dexamethasone as a corticosteroid resulted in lower mortality among those receiving mechanical ventilation (31). Psychiatric complications of corticosteroid treatment range from anxiety and insomnia to severe mood disorders, delirium and dementia (32-34). Generally, the psychiatric ADR related to high-dose corticosteroids appears 1-2 weeks after initiation of the treatment and mania, or hypomania is the most common serious reported ADR. Various forms of psychotic syndromes have also been described in corticosteroid recipients (35).

*Adverse Drug Reactions (ADR)*

The potential for neuropsychiatric ADRs lies in most of the suggested COVID-19 medications. Some of these most significant adverse effects are discussed and represented in the following segments.

*Azithromycin*

Severe adverse neuropsychiatric effects of azithromycin, such as delirium, have scarcely been published in adults (36). Nevertheless, psychotic depression, delirium, catatonia, vertigo, violent reactions, headache, anxiety, somnolence, and dizziness have been mentioned as adverse effects of azithromycin in some studies (37).

*Chloroquine and Hydroxychloroquine*

Depression, psychosis, delirium, suic-idality, agitation, mood changes, aggressive behavior, anxiety, and sleep disturbances are neuropsychiatric side effects of chloroquine and hydroxychloroquine; psychological side effects begin within a few days after starting therapy and ameliorate after ending therapy (36-38). Behavioral side effects, lightheadedness, sleep disturbance, irritability, confusional states, and psychosis are rarely reported with these drugs. In patients with malaria, traditional observations of chloroquine–induced mania have also been recorded. In patients with drug-treated rheumatoid arthritis, hydroxychloroquine sulfate has also been involved in the progression of acute psychosis, anxiety, and depression (39). Patients with COVID-19 may suffer from dyspnoea, which in turn can lead to sleeplessness, anxiety, and symptoms that may be exacerbated by CQ/HCQ (40). With doses of more than 6.5 mg/kg/day and concurrent administration of CYP3A4 inhibitors (like lopinavir/ritonavir) and glucocorticoids, the risk for HCQ is enhanced (7). Almost all evidence of chloroquine clinical adverse effects is based on case reports or case series describing symptoms such as agitation, anxiety, aggressive outbursts, depression, and suicidal ideation. It was noted that insomnia and depression were more common (41). In less than 1-2 percent of patients on chloroquine, neuropsychiatric events such as mania, depression, catatonia, and psychosis are present, and hydroxychloroquine-induced psychosis has been identified only in a few case studies. They should be assumed to be very uncommon (6). 

*Convalescent plasma therapy*

While cardiovascular complications, allergic reactions, and bronchospasm can cause symptoms like palpitations and shortness of breath that may imitate panic attacks, specific neuropsychiatric consequences have not been recorded by convalescent plasma therapy so far. A possible psychological adverse impact of convalescent plasma therapy refers to ethical concerns about the prospective donors’ coercion, secrecy, and anonymity that were originally presented during the Ebola epidemic. These issues forced the World Health Organization to offer guidelines on the ethical application of convalescent plasma (37).

*Corticosteroids*

Based on the published reports, the neuropsychiatric adverse effects of corticosteroids have been well specified, which include agitation, insomnia, mood lability, anxiety, depersonalization, delirium, depression, psychosis and manic symptoms while administered as a bolus; they could also occur in patients with COVID-19 who received corticosteroid. The majority of neuropsychiatric adverse effects typically occur within the first few days of treatment, and the most important risk factor is dosing (prednisolone equivalent dose of > 40 mg/day) (37-39). Short-term therapy with high-dose corticosteroids, as used in COVID-19, can induce mood swings and delirium, mania and hypomania, and less depression (36). It has been suggested that symptoms such as insomnia, mood changes, personality swings, major depression, and psychosis occur in 5 to 18 percent of corticosteroid-treated patients. The average prevalence of corticosteroid-related neuropsychiatric symptoms varies from 2% to 60%. Psychological changes caused by steroids vary from moderate symptoms such as insomnia, anxiety and irritability to serious symptoms such as delirium, mania, depression, and psychosis (42). In addition, the most common ADRs following short-term corticosteroid application involved euphoria and hypomania. The risk of developing mania following corticosteroid administration increases by 4 to 5 times, depending on its initial daily dose (43).

*Famotidine*

Most of the psychiatric adverse effects of famotidine in the literature were based on case reports describing symptoms such as disorientation, mental state changes with agitation, delirium, nightmares, and manic symptoms. These symptoms subsided after famotidine was discontinued. It has been suggested that these symptoms may be linked to famotidine (44-46).

*Interferon*

Interferon-alpha (IFN-α) is well-known for its depressogenic effects; Anhedonia, lack of motivation, apathy, and depressed mood are usually seen with this drug (47). Interferon-β (IFN-β) has also been linked to adverse psychological effects and other negative impacts affecting the skin, nervous system, and subcutaneous tissue (48). A meta-analysis survey demonstrated that only a wide variety of neuropsychiatric side effects are correlated with treatment with IFN-α alone or IFN-α plus ribavirin. The most prevalent and main neuropsychiatric symptom in IFN-based therapy is fatigue. Depression, anorexia, headache, mood changes, sleep disturbances were other prevalent neuropsychiatric adverse effects of IFN-α (49). An analysis of changes in mood status and biomarkers during the treatment of chronic hepatitis C with peg-interferon and ribavirin indicated that depression is a common side effect of IFN treatment in individuals suffering from chronic hepatitis C (50). IFN-α has a U.S. Food and Drug Administration (FDA) boxed warning for lethal or life-threatening neuropsychiatric conditions. These include irritability, suicidality, fatigue, anxiety disorders, lability, apathy, disrupted sleep, cognitive deficits, and mood disorders (37).

*Ivermectin*

A study on the central and peripheral nervous system disorders after ivermectin administration revealed that about 48% of individuals had significantly altered mental status. The rate of psychological conditions associated with ivermectin was 12%. Identified psychiatric disorders in these cases were abnormal behavior, agitation, and personality disorders (51). Results of another case-control study revealed that combination therapy with ivermectin and albendazole has been associated with psychosis, aggressive behavior, and disrupted sleep as well as appetite in participants (52).

*Lopinavir/Ritonavir*

Depressive symptoms have been linked with the lopinavir-ritonavir combination (36). Possible psychological side effects, including agitation, confusion, anxiety, emotional lability, and unusual dreams are also described in the manufacturer’s prescription records; however, there is inadequate data on the occurrence of such ADRs in published trials or case reports (37).

*Oseltamivir*

Oseltamivir-related psychological adverse effects like abnormal behavior have been reported (48). Several neuropsychiatric adverse events and unusual behaviors, including perceptual disturbances, depressive episodes and mania, delirium and delirium-like events, terrifying episodes, sudden anger, jumping or falling from a height and suicidal thoughts have been attributed to oseltamivir administration based on multiple studies. In March 2007, due to the potential cause of abnormal behavior, the Japanese Ministry of Health, Labour and Welfare alerted over the use of oseltamivir in children aged 10-19 years. An alert was issued to the oseltamivir label in 2006 by the FDA to attract attention to the possibility of neuropsychiatric adverse events of this agent (47).

*Remdesivir*

There is little data available on neuropsychiatric side effects of remdesivir. Although infusion-related reactions such as hypotension, diaphoresis, and shivering may occur during the administration of this drug, these symptoms may be misinterpreted as a panic attack (37). Data about the Ebola virus disease outbreak revealed that no serious neuropsychiatric adverse reactions associated with remdesivir been reported. Only one patient showed neurologic adverse reactions after remdesivir administration for Ebola treatment. Data from the COVID-19 pandemic revealed that according to a randomized controlled trial in China, remdesivir administration was not associated with the aggravation of depression and schizophrenia in COVID-19 patients. Also, a multi-center open-label cohort study in COVID-19 patients from Canada, the USA, Japan, and Europe revealed that delirium was recorded in 2 out of 61 patients receiving remdesivir. Finally, a recent study on remdesivir therapy in COVID-19 patients has reported no significant psychiatric and/or neurologic adverse reactions (53).

*Ribavirin*

As reported in some studies, the use of ribavirin in combination with either interferon or direct-acting antiviral agents may be associated with an increased incidence of psychiatric disturbances compared to monotherapy. These include depressed mood, irritability, anxiety, sleep disturbances, and sexual dysfunction. Because ribavirin is commonly used in combination with IFN or direct-acting antivirals in clinical practice, it is not clear whether these ADRs can be attributable to ribavirin alone (47). 

*Tocilizumab*

Since pain, fatigue, depression, and anemia are significantly associated with IL-6, the administration of tocilizumab as an IL-6 antagonist would be promising in the alleviation of these signs/symptoms (54). Also, recent findings from patients with rheumatoid arthritis have indicated that tocilizumab may have some beneficial effects on depressive and anxiety disorders (55).

*Potential Drug-Drug Interactions (PDDIs)*

Potential drug-drug interactions (PDDIs) of COVID-19 treatment options and psychotropic medications are listed in Table 1. The most common major DDIs are attributed to chloroquine, hydroxychloroquine, azit-hromycin, unfractionated heparin (UFH) and enoxaparin with antidepressant agents including selective serotonin reuptake inhibitors (SSRIs) and serotonin-norepinephrine reuptake inhibitors (SNRIs). Also, lopinavir/ritonavir, chloroquine, hydroxychloroquine, and azithromycin could induce major DDIs with antidepressant agents (*e.g*., trazodone, nefazodone, and vilazodone), many typical and atypical antipsychotic agents, benzodiazepines with active metabolites (*e.g*., alprazolam, clonazepam, chlordiazepoxide), and mood stabilizers (*e.g*., lithium, carbamazepine). It seems that among psychotropic medications discussed here, carbamazepine, haloperidol, pimozide, and ziprasidone may have the highest incidence of PDDIs with COVID-19 treatment options. So, drug selection and prescription should be performed with caution in psychiatric patients who have been infected with SARS-CoV-2 in order to avoid major DDIs and subsequent clinical complications. 

Potential mechanisms of major DDIs are listed in Table 2. Also, Up-To-date® and Liverpool® recommendations on interaction management are discussed in this table. The most common type of major PDDIs is pharmacokinetics. Hydroxychloroquine/chloroquine and lopinavir/ritonavir were the most involved anti-COVID-19 agents in these major PDDIs.

**Table 1:** Potential Drug-Drug Interactions of COVID-19 treatment options with Psychotropic Medications

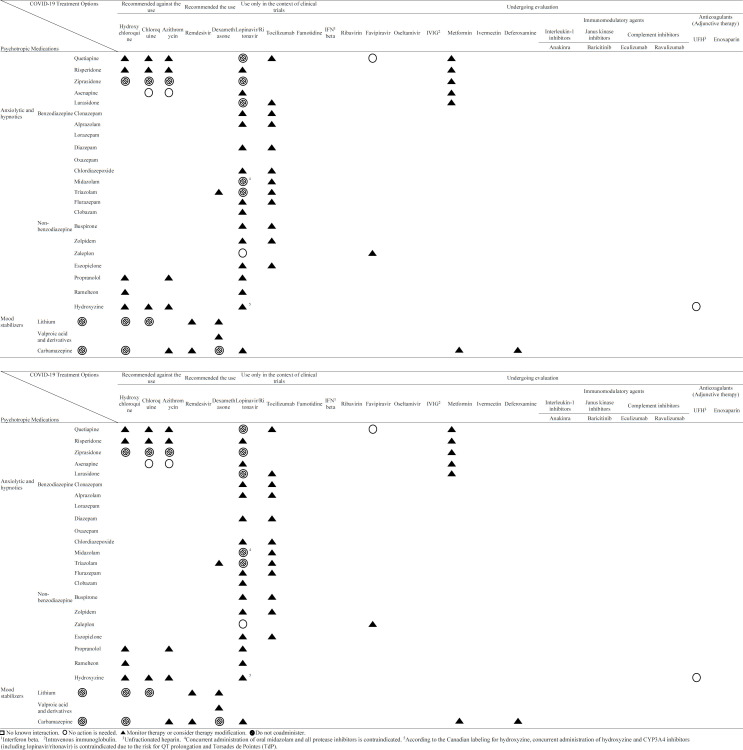

**Table 2:** Possible mechanisms and managements of major drug-drug interactions among anti-COVID-19 and psychotropic medications

**Medicaments**	**Type of interaction**	**Mechanism of Interaction**	**Interaction Management**
Hydroxychloroquine or Chloroquine plus SSRI (Citalopram or Escitalopram) or Haloperidol or Pimozide or Ziprasidone or Lithium	Pharmacodynamic	The QTc-prolonging effect of hydroxychloroquine (or chloroquine) may be enhanced by QT-prolonging medications.	Do not coadminister.
Hydroxychloroquine or Chloroquine plus Venlafaxine	Pharmacokinetic and Pharmacodynamic	Hydroxychloroquine (or chloroquine) by inhibiting CYP2D6 may increase the concentration of venlafaxine and thus increase the risk of QT-prolongation. Both drugs have a risk of QT-prolongation.	Do not coadminister.
Hydroxychloroquine or Chloroquine plus Clomipramine	Pharmacokinetic and Pharmacodynamic	Hydroxychloroquine (or chloroquine) by inhibiting CYP2D6 may increase the concentration of clomipramine and thus increase the risk of QT-prolongation. Both drugs have a risk of QT-prolongation.	Do not coadminister.
Hydroxychloroquine or Chloroquine plus Thioridazine	Pharmacokinetic and Pharmacodynamic	Hydroxychloroquine (or chloroquine) may increase the concentration of thioridazine by inhibiting CYP2D6. Thioridazine may reduce the concentration of hydroxychloroquine or chloroquine by inducing CYP3A4 (moderate inducer). Both drugs have a risk of QT-prolongation.	Do not coadminister.
Hydroxychloroquine or Chloroquine plus Carbamazepine	Pharmacokinetic	Carbamazepine may induce hydroxychloroquine (or chloroquine) metabolism, leading to a significant reduction in plasma concentration.	Do not coadminister.
Azithromycin plus Citalopram or Escitalopram or Haloperidol or Pimozide or Venlafaxine or Lithium or Clomipramine or Ziprasidone or Thioridazine	Pharmacodynamic	The QTc-prolonging effect of azithromycin may be enhanced by QT-prolonging medications.	Do not coadminister.
Lopinavir/ritonavir plus Midazolam	Pharmacokinetic	The midazolam serum concentration may be increased by protease inhibitors.	Do not coadminister. It is contraindicated to co-administer **oral** midazolam with lopinavir/ritonavir. Concomitant use of intravenous midazolam with lopinavir/ritonavir should only be done with caution and in a setting that ensures accurate monitoring and medical management of any excessive response to this drug. In concomitant use, dosage reduction for intravenous midazolam should be considered, especially in multiple doses.
Lopinavir/ritonavir plus Triazolam	Pharmacokinetic	Lopinavir/ritonavir (strong CYP3A4 inhibitor) may increase the triazolam serum concentration.	Do not coadminister.
Lopinavir/ritonavir plus Diazepam	Pharmacokinetic	Lopinavir/ritonavir (strong CYP3A4 inhibitor) may increase the diazepam serum concentration.	Consider therapy modification. Due to the increase in diazepam concentration and sedative effects, it may be necessary to reduce the dose of diazepam if co-administered with Lopinavir/ritonavir.
Lopinavir/ritonavir plus Lurasidone	Pharmacokinetic	Lopinavir/ritonavir (strong CYP3A4 inhibitor) may increase the lurasidone serum concentration.	Do not coadminister.
Lopinavir/ritonavir plus Pimozide	Pharmacokinetic	Lopinavir/ritonavir (strong CYP3A4 inhibitor) may increase the pimozide serum concentration.	Do not coadminister.
Lopinavir/ritonavir plus Ziprasidone	Pharmacokinetic and Pharmacodynamic	Lopinavir/ritonavir (strong CYP3A4 inhibitor) may increase the ziprasidone serum concentration. Both drugs have a risk of QT-prolongation.	Do not coadminister.
Lopinavir/ritonavir plus Quetiapine	Pharmacokinetic and pharmacodynamic	Lopinavir/ritonavir (strong CYP3A4 inhibitor) may increase the quetiapine serum concentration. Both drugs have a risk of QT-prolongation.	Do not coadminister. According to European labeling, co-administration of quetiapine with CYP3A4 inhibitors is contraindicated. The American labeling recommends that in case of concomitant use with a strong CYP3A4 inhibitor, the quetiapine dose should be reduced to one-sixth of the regular dose
Lopinavir/ritonavir plus Haloperidol	Pharmacokinetic and pharmacodynamic	Lopinavir/ritonavir (strong CYP3A4 inhibitor) may increase the haloperidol serum concentration. Both drugs have a risk of QT-prolongation.	Do not coadminister.
Lopinavir/ritonavir plus Aripiprazole	Pharmacokinetic and pharmacodynamic	Lopinavir/ritonavir (strong CYP3A4 inhibitor) may increase the aripiprazole serum concentration. Both drugs have a risk of QT-prolongation.	Consider therapy modification. European labeling recommends reducing the aripiprazole dose to 50% of its usual dose when given with strong CYP3A4inhibitors. Monitor adverse effects.
Lopinavir/ritonavir plus Carbamazepine	Pharmacokinetic	lopinavir/ritonavir (strong CYP3A4 inhibitor) may increase the carbamazepine serum concentration. Carbamazepine (CYP3A4 inducer) may reduce Lopinavir serum concentration.	Do not coadminister. Lopinavir/ritonavir once-daily regimen should not be used with carbamazepine. Appropriate exposure to lopinavir/ritonavir may be obtained at higher doses of lopinavir/ritonavir (400/400mg twice daily) associated with an increased risk of liver and gastrointestinal toxicity.
Lopinavir/ritonavir plus Buspirone	Pharmacokinetic	Lopinavir/ritonavir (strong CYP3A4 inhibitor) may increase the buspirone serum concentration.	Consider therapy modification. When co-administered with lopinavir/ritonavir, the dose of buspirone should be limited to 2.5 mg per day, and the patient should be monitored for increased effects of buspirone.
Lopinavir/ritonavir plus Trazodone	Pharmacokinetic and pharmacodynamic	Lopinavir/ritonavir (strong CYP3A4 inhibitor) may increase the trazodone serum concentration. Both drugs have a risk of QT-prolongation.	Consider therapy modification. Concomitant use of trazodone and lopinavir/ritonavir should be done with caution. A lower dose of trazodone and monitoring for increased effects of trazodone such as QT-prolongation and sedation should be considered.
Lopinavir/ritonavir plus Nefazodone	Pharmacokinetic	Lopinavir/ritonavir (strong CYP3A4 inhibitor) may increase the Nefazodone serum concentration.	Consider therapy modification. In patients treated with lopinavir/ritonavir, an alternative drug to nefazodone or a dose reduction should be considered.
Lopinavir/ritonavir plus Vilazodone	Pharmacokinetic	lopinavir/ritonavir (strong CYP3A4 inhibitor) may increase the vilazodone serum concentration.	Consider therapy modification. Limit the maximum daily dose of vilazodone to 20 mg if coadministered with lopinavir/ritonavir.
Unfractionated Heparin plus SSRIs (Fluoxetine or Paroxetine or Citalopram or Escitalopram or Sertraline or Fluvoxamine) or SNRIs (Venlafaxine or Duloxetine) or Milnacipran or Vilazodone or Vortioxetine	Pharmacodynamic	The anticoagulant effect of heparin may be enhanced by agents with antiplatelet properties.	Consider therapy modification. If coadministration is required, reduce the dose of heparin or antiplatelet agents.
Enoxaparin plus SSRIs (Fluoxetine or Paroxetine or Citalopram or Escitalopram or Sertraline or Fluvoxamine) or SNRIs (Venlafaxine or Duloxetine) or Milnacipran or Vilazodone or Vortioxetine	Pharmacodynamic	The anticoagulant effect of Enoxaparin may be enhanced by agents with antiplatelet properties.	Consider therapy modification. Before initiating Enoxaparin discontinue antiplatelet agents whenever possible. Monitor patients for signs and symptoms of bleeding if coadministration is inevitable.
Dexamethazone plus Carbamazepine	Pharmacokinetic	Carbamazepine (strong CYP3A4 inducers) may induce the metabolism of systemic dexamethasone and decrease its serum concentration.	Consider therapy modification. Increase the dose of dexamethasone in patients receiving carbamazepine and monitor for reduced dexamethasone efficacy. In the treatment of life-threatening conditions, if possible, avoid this combination.

## Conclusion

SARS-CoV-2 can lead to psychiatric complications, with biological and psychosocial factors may contribute to its pathogenesis, as well as COVID-19 medications can cause psychiatric symptoms. On the other hand, concomitant use of COVID-19 medications and psychotropics can lead to PDDIs that may culminate in serious and life-threatening conditions. Psychiatric aspects of COVID-19 and its treatment options, knowledge of the pharmacokinetics of COVID-19 medications as well as psychotropics, and the management of possible interactions of these drugs are of considerable importance in managing patients with COVID-19.

## Author Contributions

All authors made substantial contributions to conception and design, acquisition of data, or analysis and interpretation of data; took part in drafting the article or revising it critically for important intellectual content; gave final approval of the version to be published; and agree to be accountable for all aspects of the work.
